# Dengue and Zika Virus Capsid Proteins Contain a Common PEX19-Binding Motif

**DOI:** 10.3390/v14020253

**Published:** 2022-01-27

**Authors:** Mafalda A. Farelo, Despoina Korrou-Karava, Katrina F. Brooks, Tiffany A. Russell, Kevin Maringer, Peter U. Mayerhofer

**Affiliations:** 1 School of Biosciences and Medicine, Faculty of Health and Medical Sciences, University of Surrey, Guildford GU2 7XH, UK; mafaldad@ucr.edu (M.A.F.); dk00324@surrey.ac.uk (D.K.-K.); kb00400@surrey.ac.uk (K.F.B.); tiff_russell@outlook.com (T.A.R.); 2 The Pirbright Institute, Pirbright GU24 0NF, UK

**Keywords:** peroxisomes, human peroxin PEX19, flavivirus, dengue virus, Zika virus, capsid protein

## Abstract

Flaviviruses such as dengue virus (DENV) and Zika virus (ZIKV) have evolved sophisticated mechanisms to suppress the host immune system. For instance, flavivirus infections were found to sabotage peroxisomes, organelles with an important role in innate immunity. The current model suggests that the capsid (C) proteins of DENV and ZIKV downregulate peroxisomes, ultimately resulting in reduced production of interferons by interacting with the host protein PEX19, a crucial chaperone in peroxisomal biogenesis. Here, we aimed to explore the importance of peroxisomes and the role of C interaction with PEX19 in the flavivirus life cycle. By infecting cells lacking peroxisomes we show that this organelle is required for optimal DENV replication. Moreover, we demonstrate that DENV and ZIKV C bind PEX19 through a conserved PEX19-binding motif, which is also commonly found in cellular peroxisomal membrane proteins (PMPs). However, in contrast to PMPs, this interaction does not result in the targeting of C to peroxisomes. Furthermore, we show that the presence of C results in peroxisome loss due to impaired peroxisomal biogenesis, which appears to occur by a PEX19-independent mechanism. Hence, these findings challenge the current model of how flavivirus C might downregulate peroxisomal abundance and suggest a yet unknown role of peroxisomes in flavivirus biology.

## 1. Introduction

Flaviviruses such as yellow fever virus (YFV), West Nile virus (WNV), Zika virus (ZIKV), and dengue virus (DENV) are a significant group of arthropod-transmitted viral pathogens. Due to urbanization, climate change, and global travel, these viruses pose an increasing threat to global public health. Even though infections may be severe or fatal, specific antiviral therapies are not available. Vaccinations for many flaviviruses are not developed or, for DENV, not suitable for use in all settings. Thus, treatments are limited to symptomatic and supportive care [1,2].

Peroxisomes are metabolic organelles that are essential for life. Peroxisomes are responsible for very-long chain fatty acids beta-oxidation, ether phospholipid and bile acid biosynthesis, and cellular redox metabolism [3]. Their medical relevance is underscored by the fact that genetic disorders that lead to complete peroxisomal biogenesis failure result in early-lethal human diseases, such as Zellweger-syndrome [4,5]. In addition to these essential metabolic functions, recent studies found peroxisomes to be important platforms for innate immunity. The mitochondrial antiviral-signaling protein (MAVS, also known as IPS-1, Cardif, or VISA), a membrane protein with a key role in retinoic acid inducible gene I (RIG-I)-like receptor (RLR)-dependent interferon (IFN) induction, was found not only to localize to mitochondria, but also in peroxisomal membranes [6]. MAVS has also been shown to play a central role in the peroxisome-dependent immune response [6,7].

Multiple viruses, including flaviviruses, have developed strategies to modulate peroxisome numbers either to exploit their role in lipid metabolism or to escape the peroxisome-dependent innate immune response (a topic recently reviewed by Ferreira et al. [8]). For instance, peroxisomes have a key function in supporting Kaposi’s sarcoma associated herpesvirus (KSHV; *Human gammaherpesvirus 8*) latency by stabilizing the peroxisome-localized viral FADD-like interleukin-1-β–converting enzyme-inhibitory protein (vFLIP), which is a viral oncoprotein [9]. Other viruses directly sabotage peroxisome-mediated immune signaling pathways. The NS3/4A protease of hepatitis C virus (HCV) cleaves mitochondrial as well as peroxisomal MAVS, which results in suppressed activation of the IFN response [10,11,12]. Certain viral proteins interfere with IFN signaling downstream of peroxisomal MAVS, such as the human cytomegalovirus (HCMV) protein viral mitochondria-localized inhibitor of apoptosis (vMIA) [13], or herpes simplex virus type 1 (HSV-1; *Human alphaherpesvirus 1*) tegument protein VP16 [14]. A different strategy is applied by human immunodeficiency virus (HIV-1), which upregulates a subset of cellular miRNAs that suppress the expression of several peroxisomal biogenesis genes. Thus, peroxisome numbers are reduced during acute as well as chronic HIV-1 infection [15]. Severe acute respiratory syndrome-related coronavirus 2 (SARS-CoV-2) infection also alters peroxisome homeostasis, as it reduces the number of mature peroxisomes, likely through the impairment of peroxisomal protein import machinery due to an interaction between the peroxisomal protein PEX14 and the viral protein ORF14 [16].

Within the *Flavivirus* genus, a significant loss of peroxisomes was observed during DENV, WNV and ZIKV infection [17,18,19], which was suggested to be mediated through an interaction between the flavivirus capsid (C) protein and PEX19, a peroxisomal biogenesis chaperone with a key function in early peroxisomal membrane biogenesis.

PEX19 recognizes and binds peroxisomal membrane proteins (PMP), which are translated on free polyribosomes in the cytosol. In general, the PEX19-cargoPMP interaction requires both a PEX19 binding consensus motif and a hydrophobic α-helical transmembrane segment in close proximity. These PEX19 binding motifs are α-helical regions composed of basic amino acids, which are surrounded by hydrophobic amino acids that are placed in a defined distance to each other [20,21,22,23]. Hence, PEX19 acts as PMP receptor [24] and chaperone [25], thereby keeping PMPs in a membrane-insertion competent conformation. Subsequently, PEX19 targets the PMPs to the peroxisome, where it mediates their insertion into the organelle bilayer [26]. The current model suggests that flavivirus (WNV, DENV, and ZIKV) C proteins sequester PEX19, thereby blocking its cellular function in peroxisomal biogenesis, resulting in peroxisome loss and subsequent dampening of IFN signaling, facilitating enhanced viral replication. However, data also indicate that decrease or loss of peroxisomes leads to an impairment in flavivirus replication [17,18], which contradicts the hypothesis that the peroxisomal immune response suppresses flavivirus replication. Thus, ZIKV replication has been shown to be impaired in peroxisome-deficient patient-derived human fibroblasts [18] and DENV and WNV replication is reduced in cells where PEX19, a crucial chaperone in peroxisome biogenesis, is transiently reduced using siRNAs [17].

Here, we aimed to further understand the importance of peroxisomes in flavivirus replication and to explore the role of the interaction between PEX19 and flavivirus C proteins. We also characterize the molecular mechanism behind the C-PEX19 interaction. We show that DENV replication is impaired in human patient-derived fibroblasts lacking peroxisomes, highlighting the importance of peroxisome interactions to DENV replication. Additionally, we demonstrate that DENV and ZIKV C proteins contain a conserved PEX19-binding motif, which is common to cellular PMPs. However, unlike PMP substrates of PEX19, this PEX19-binding motif is not an integral part of a peroxisomal targeting signal, as the C proteins do not localize to peroxisomes. Furthermore, we found that the presence of C proteins results in a loss of peroxisomes due to impaired peroxisome biogenesis, which occurs by a PEX19-independent mechanism. These findings highlight the importance of peroxisomes in flavivirus replication and challenge the current model of how flavivirus C proteins downregulate peroxisomal abundance.

## 2. Methods

### 2.1. Cloning and Plasmids

DENV (DENV-2, GI:323448) and ZIKV (isolate Paraiba 01, KX280026) C proteins, and ALD, were synthesized de novo as DNA fragments and thereafter used as a template for PCR amplification. PCR reactions were performed under standard conditions using Phusion DNA polymerase (Thermo Fisher Scientific, Waltham, MA, USA) and synthetic oligonucleotide primers (sequences are available on request). All constructs were verified by DNA sequencing. For expression in human cells, PCR-generated products were inserted into pIRES2-EGFP (Takara Biosciences, Mountain View, CA, USA) via the respective restriction sites upstream of the internal ribosome entry site (IRES) and enhanced GFP. For the construct ZIKV C^flag^, a PCR fragments containing the ZIKV C cDNA (including residues 1–104), a *Pst*I site, and the coding sequence for triple flag tag (DYKDHDGDYKDHDIDYKDDDDK), was cloned into the pIRES2-EGFP plasmid using *EcoR*I and *BamH*I sites, respectively. This construct was then used to generate DENV C^flag^ by replacing the ZIKV cDNA with the DENV cDNA (including residues 1–104), using *Nhe*I and *Pst*I sites, respectively. For the construct ALD^flag^, the ZIKV C sequence was replaced with the ALD cDNA (including residues 1–166), using *Nhe*I and *Pst*I sites. For mutations within and outside the PEX19-binding motif, residues R23V24, K31R32, K45L46 (DENV), R45M46 (ZIKV), R55F56, and R69V70 and R80L81 within ALD were simultaneously replaced by alanine-glycine (introducing a *Nae*I site) by site-directed mutagenesis using standard conditions and the pIRES2-EGFP plasmids DENV C^flag^, ZIKV C^flag^, and ALD^flag^ as templates. The plasmid pIRES2-EGFP/ICP47^flag^ was described previously [27]. The pIRES2-EGFP derived plasmids encoding for ZIKV/DENV C^flag^ (WT and variants); ALD^flag^, or ICP47^flag^ were then used to subclone each flag-tagged insert into the plasmid pcDNA3.1(+) using *Nhe*I and *BamH*I sites. To generate the plasmid pIRES2-mCherry-SKL/PEX19, the PEX19 cDNA was amplified by PCR (using the plasmid pcDNA3/PEX19 all as template [28]), and subsequently cloned into pIRES2-EGFP using *Sal*I and *BamH*I sites, generating the plasmid pIRES2-EGFP/PEX19. Thereafter, the EGFP CDS was replaced with a PCR fragment coding for mCherry-SKL (which contains a C-terminal peroxisomal matrix targeting signal ‘SKL’ [29]) using *BstX*I and *Not*I sites. Accordingly, pIRES2-mCherry-SKL was generated by replacing the EGFP CDS of pIRES2-EGFP with a PCR fragment encoding for mCherry-SKL using *BstX*I and *Not*I sites. To generate pIRES2-mCherry-SKL/PEX3, the PEX3 cDNA was amplified by PCR (using the plasmid pcDNA3.1/Pex3-Myc-His as template [5]) and cloned into pIRES2-mCherry-SKL using *Nhe*I and *Sac*I sites. pNoGFP-EGFP-SBP-PEX19 was generated by replacing the EGFP CDS of the plasmid pEGFP-N3 with a synthetic DNA fragment encoding for an EGFP-Streptavidin Binding Peptide (SBP)-PEX19 fusion protein using *Xho*I and *Not*I sites.

### 2.2. Cell Lines, and Transfection

HEK293T (human embryonic kidney cells), A549 (lung carcinomatous cells), HFFF (human foreskin fibroblast cells), *PEX19*-deficient [4] and *PEX3*-deficient [5] (human skin fibroblast) cell lines were maintained in Dulbecco’s modified Eagle’s medium supplemented with 10% fetal bovine serum at 37 °C in a 5% CO_2_-humidified atmosphere. BHK (baby hamster kidney) and C6/36 (*Aedes albopictus* mosquito cells) were kind gifts from Ana Fernandez-Sesma (Icahn School of Medicine at Mount Sinai, New York, NY, USA). BHK cells were maintained in minimal essential medium (α-MEM) supplemented with 10% fetal bovine serum, 10 mM HEPES, 100 units per mL (U/mL) penicillin and 100 µg/mL streptomycin at 37 °C in a 5% CO_2_-humidified atmosphere. C6/36 cells were maintained in Roswell Park Memorial Institute medium supplemented with 10% fetal bovine serum, 2 mM L-glutamine, 1 mM sodium pyruvate, 0.1 mM non-essential amino acids, 0.15% sodium bicarbonate, 100 U/mL penicillin and 100 µg/mL streptomycin at 37 °C in a 5% CO_2_-humidified atmosphere.

For streptavidin pull-down or co-immunoprecipitation assays, HEK293T cells were seeded in 10 cm^2^ dishes with a density of 3 × 10^6^ cells/mL. The next day, cells were transfected using 30 μg of polyethyleneimine (Sigma-Aldrich, St. Louis, MO, USA) and 10 μg of plasmid DNA according to standard procedures. For immunofluorescence, A549 or *PEX19*-deficient cells were seeded in 6-well plates with a density of 2 × 10^5^ cells/well the day before transfection. Transient transfection was conducted according to the Magnetofection protocol (Chemicell, Berlin, Germany) using 2 μg of plasmid DNA and 2 μL of PolyMAG (Chemicell) per well.

### 2.3. Virus and Infections

DENV-2 strain 16681 was a kind gift from Ana Fernandez-Sesma. Virus stocks were prepared in C6/36 cells and titrated on BHK cells as described previously [30]. For fibroblast infection experiments, confluent HFFF, *PEX19*-deficient and *PEX3*-deficient cell lines grown in 24-well plates were infected at an MOI of 2 or 0.02 one day post-seeding by removing the culture supernatant and incubating for one hour with viral inoculum in sterile PBS with periodic rocking, after which the inoculum was removed and replaced with culture medium. Cells were incubated at 37 °C in a 5% CO_2_-humidified atmosphere and supernatants harvested at the time points indicated, stored at −80 °C and titrated on BHK cells as previously described [30].

### 2.4. Streptavidin Affinity Purification, Immunoprecipitation, and Immunoblotting

24 h after transfection, HEK293T cells were washed twice in PBS, followed by lysis and solubilization in NP-40 lysis buffer [50 mM Tris/HCl pH 7.4, 150 mM NaCl, 2 mM EDTA, 1% (*v*/*v*) Nonidet P-40 (Sigma-Aldrich, St. Louis, MO, USA), 1% protease inhibitor cocktail III (Thermo Fisher Scientific)] for 1 h at 30 min at 4 °C. Non-solubilized proteins were removed by centrifugation at 17,000× *g* for 30 min at 4 °C. The supernatant was then used either for streptavidin affinity purification, or immunoprecipitation experiments. For affinity purification, 50 μL streptavidin agarose beads (Thermo Fisher Scientific) were washed once in H_2_O, and once in NP-40 lysis buffer, following incubation with the supernatant for 1 h (or overnight) at 4 °C. The beads were then washed three times with 1 mL NP-40 lysis buffer. Proteins were eluted either with NP-40 lysis buffer supplemented with 2.5 mM biotin, or 2× SDS-PAGE sample buffer (4% SDS, 100 mM Tris/HCl, pH 8.0, 400 mM DTT, 20% glycerol, 0.1% bromo-phenol blue) for 10 min at 80 °C. For immunoprecipitation, the supernatant was incubated for 1 h (or overnight) at 4 °C with Protein G Dynabeads (Thermo Fisher Scientific), which had been pre-loaded with anti-PEX19 antibodies (rabbit monoclonal, ab137072; Abcam, Cambridge, UK) or unrelated antibodies as negative control (anti-PEX3 rabbit, Sigma-Aldrich HPA042830; anti-HA rabbit, PRB-101P; Biolegend, San Diego, CA, USA) according to the manufacturer instructions. Beads were washed three times with NP-40 lysis buffer and eluted in 2× SDS-buffer for 10 min at 80 °C. Prior to immunoblotting, samples were denatured for 10 min at 80 °C and separated by SDS-PAGE. After transfer onto nitrocellulose membranes, proteins were detected with anti-flag (mouse monoclonal Anti-flag M2, Sigma-Aldrich) or anti-PEX19 (mouse monoclonal, Santa Cruz, Dallas, TX, USA) specific antibodies as indicated. Horseradish peroxidase (HRP) conjugated secondary antibodies were detected with the SuperSignal West Pico Kit (Thermo Fisher Scientific) and Fusion X imaging system (Sigma-Aldrich). One representative blot out of at least three independent experiments is shown.

### 2.5. Immunofluorescence and Image Processing

Transiently transfected A549 cells or PEX19-deficient fibroblasts grown on cover slips were washed in PBS, fixed for 30 min with 3.7% formaldehyde in PBS at room temperature, and permeabilized with 1% Triton X-100 for 5 min. Following one washing step in PBS, cells were incubated for 1 h with primary antibodies against flag (mouse monoclonal Anti-flag M2, Sigma-Aldrich, 1:1000 in PBS), catalase (goat polyclonal, Bio-Techne, Minneapolis, MN, USA, 1:50 in PBS), or PMP70 (mouse monoclonal, Sigma-Aldrich, 1:500 in PBS). The cells were then washed 5 times with PBS and incubated for 1 h in the dark with secondary antibodies: Donkey anti-Mouse Alexa Fluor 488 (Thermo Fisher Scientific, Waltham, MA, USA, 1:2000 in PBS), Chicken anti-Goat Alexa Fluor 647 (Thermo Fisher Scientific, 1:500 in PBS), or Goat anti-Mouse Alexa Fluor 594 (Thermo Fisher Scientific, 1:1000 in PBS). After 10 wash steps in PBS, the cells were embedded in an antifading reagent (Vectashield, Vector Laboratories Inc., Burlingame, CA, USA) with or without DAPI. Lipid droplets were stained by LipidTOX deep red (Thermo Fisher Scientific) according to manufacturer’s instructions. Fluorescence microscopy was performed using a Nikon A1M Eclipse Ti confocal microscope equipped with a 60× oil lens. Two-dimensional square images were taken using either at line average of 8× or 16× and frame size of 512 or 1024 pixels. The NIS-Elements Advanced Research software version 4.50 (Nikon Instruments, Melville, NY, USA) was used for Z-stacks acquisition (images spaced by 0.2 μm), and colocalization analysis (Pearson correlation coefficient).

## 3. Results

### 3.1. DENV Replication Is Impaired in the Absence of Peroxisomes

**Figure 1:**
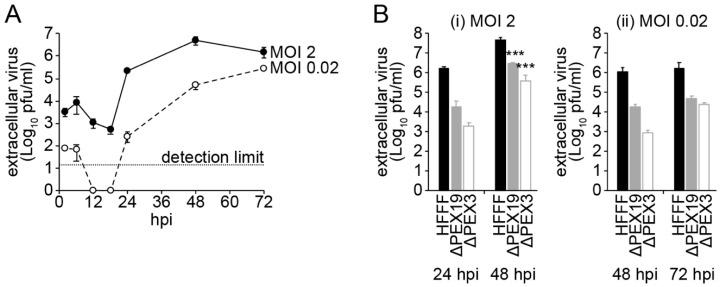
Peroxisomes are required for optimal DENV replication. (**A**) HFFF cells were infected with DENV-2 at an MOI of 2 or 0.02 and the appearance of infectious virus in the extracellular supernatant quantified by plaque assay over a 72-h time course of infection. (**B**) Primary human fibroblasts (HFFF) and *PEX19*-deficient (∆PEX19) or *PEX3*-deficient (∆PEX3) patient-derived fibroblasts were infected with DENV-2 at an MOI of 2 or 0.02 and extracellular infectious virus quantified by plaque assay at the indicated time points. Graphs show mean +/− standard deviation. pfu, plaque-forming units. *** *p* < 0.001 (two-way ANOVA, Tukey correction for multiple comparisons).

Since the influence of peroxisomes on DENV replication has only been tested using transient siRNA-mediated silencing of PEX19 [17], we set out to measure the impact of complete peroxisomal loss on DENV replication using an established patient-derived fibroblast model. First, we assessed the replication kinetics of DENV-2 in primary human foetal foreskin fibroblasts (HFFF) by performing single-step and multi-step growth curves in HFFF cells (Figure 1A). At a high multiplicity of infection (MOI 2), DENV-2 replication peaked 48 h post-infection (hpi). Meanwhile, DENV-2 replication had not yet plateaued 72 hpi in a multi-step growth curve (MOI 0.02). We next infected HFFF cells (serving as our ‘wild-type’ control) and established *PEX19*-deficient [4] and *PEX3*-deficient [5] patient-derived human fibroblasts at MOI 2 and 0.02 and analysed viral titres at three different time-points. Both cell lines completely lack peroxisomes due to mutations in proteins that are crucial in PMP import machinery. *PEX19*-deficient cells lack a functional PEX19 chaperone, which precludes the transport of PMPs, including PEX3, to peroxisomes while *PEX3*-deficient cells lack PEX3, precluding PMP import into peroxisomal membranes, but have a functional PEX19 [4,5]. We observed a 1–3 log reduction in DENV-2 replication at all time points tested during both high and low MOI infection in both peroxisome-deficient cell lines (Figure 1B). The differences between the PEX19- and PEX3-deficient cell lines are not statistically significant (two-way ANOVA; Tukey correction for multiple comparisons). These results suggest that peroxisomes are required for optimal DENV replication and reinforce earlier reports of an important role of peroxisomes in the life cycle of flaviviruses.

### 3.2. DENV Capsid Protein Binds PEX19 via A PEX19-Binding Motif

Previous research suggested that interaction between DENV C and PEX19 is a mechanism of antiviral antagonism, which would ultimately benefit viral replication. However, our results (Figure 1B) show that the converse is true, and that DENV replication is reduced by a lack of peroxisomes or PEX19. Thus, we decided to further explore the interaction between DENV C and PEX19, and its functional relevance by first revealing the underlying molecular determinants underpinning the interaction.

**Figure 2:**
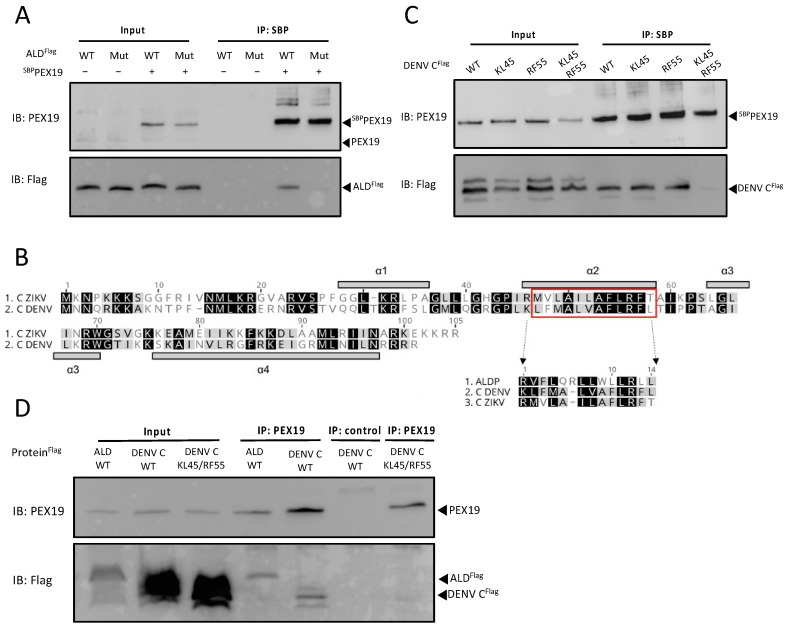
DENV C protein binds PEX19 via a PEX19-binding motif. (**A**) ^SBP^PEX19 was co-expressed in HEK293T cells together with ALD^flag^ WT or with ALD^flag^ harbouring mutations R69A, V70G, R80A, and L81G within its PEX19-binding motif (Mut^.^). ^SBP^PEX19-DENV C^flag^ complexes were affinity purified using streptavidin agarose bead and analyzed by immunoblotting using PEX19- or flag-specific antibodies, respectively. (**B**) Sequence alignment of ZIKV and DENV C proteins. The PEX19 binding motif is boxed in red, and the four alpha helices (a1–a4) [32] are indicated by grey boxes. (**C**) ^SBP^PEX19 was co-expressed in HEK293T cells together with the DENV C WT construct or DENV C with mutations within the predicted PEX19-binding motif (KL45, RF55, or both). ^SBP^PEX19-DENV C^flag^ complexes were affinity purified using streptavidin agarose beads and analyzed by immunoblotting using PEX19- or flag-specific antibodies, respectively. (**D**) ALD^flag^, the DENV C^Flag^ WT, or mutant version (KL45/RF55) were expressed in HEK293T cells. Protein complexes were affinity purified using PEX19-specific antibodies. An unrelated antibody was used as negative control (control). Samples were subsequently analyzed by immunoblotting using PEX19- or flag-specific antibodies, respectively. The amount loaded for input represents 15% of the amount loaded for the IP.

The use of GST-pull down assays for the study of PEX19 interaction with other proteins is well established [24]. However, GST is a large tag (26 kDa) when compared to DENV C (15 kDa) and therefore it may hamper interaction between PEX19 and C. Hence, we developed a new PEX19 pull-down assay using a smaller peptide-tag. A streptavidin binding peptide (SBP, 5 kDa) was fused to the N-terminus of PEX19 (^SBP^PEX19), in order to preserve the functionally important farnesylation site (CaaX motif) at the extreme C-terminus of PEX19 [31]. To assess the performance of this new approach we used a truncated version of human adrenoleukodystrophy protein (ALD), a PMP and well-characterized PEX19 interactor [24], as a positive control. This truncated version of ALD includes amino acids 1–166, which contains its first two transmembrane segments, and has been previously reported to maintain its ability to interact with PEX19 [20]. We will refer to this truncated ALD protein as “ALD” through this manuscript. HEK293T cells were transiently transfected to express flag-tagged ALD (ALD^flag^) or flag-tagged DENV C (DENV C^flag^) alone or in combination with ^SBP^PEX19. Cells were lysed and protein complexes were affinity purified using streptavidin agarose beads. ALD was successfully co-precipitated along with ^SBP^PEX19, but not in the absence of ^SBP^PEX19, confirming that this system can identify PEX19 interactions (Figure 2A). Hence, SBP-pull down is an alternative and applicable way to study protein interactions with PEX19.

An interaction between DENV C and PEX19 was recently reported [17]. We therefore determined whether the C proteins might contain a PEX19-binding motif using the BLOCKS algorithm from the PeroxisomeDB database [33]. Interestingly, we identified a single putative PEX19-binding motif that includes amino acids 46 to 57 of DENV C and ZIKV C (Figure 2B). This putative PEX19-binding motif is relatively similar to PEX19-binding motifs of other well-known PEX19 substrates [22], such as the ALD protein [24] (Figure 2B). However, unlike canonical PEX19 substrates, flavivirus mature C proteins do not possess a hydrophobic α-helical transmembrane segment in close proximity to the PEX19-binding motif. Thus, we aimed to reveal the underlying molecular basis of the interaction between PEX19 and mature flavivirus C proteins that lack a transmembrane segment. First, we used our streptavidin pulldown assay to confirm the recently reported interaction between PEX19 and DENV C (Figure 2C).

Next, we performed site-directed mutagenesis of conserved residues within the predicted PEX19-binding motifs to assess their functional importance for PEX19 binding, a strategy that has been successfully applied to inhibit the PEX19-cargo interaction of several physiological PMP substrates of PEX19 [22,23,25]. We first used the streptavidin pull-down assay to assess whether such mutations would interfere with the interaction between ALD and PEX19. By replacing four of the conserved residues (either basic or hydrophobic amino acids) within the PEX19-binding motif of ALD by alanine or glycine (ALD^flag^ Mut^.^: R69A, V70G, R80A, L81G), a mutated variant with a disturbed PEX19-binding motif was generated. Pull-down experiments revealed that only ALD^flag^ WT, but not the mutated variant (ALD^flag^ Mut), which lacks key conserved residues, was able to form complexes with PEX19 (Figure 2A). An identical approach was applied to DENV C, to assess whether the corresponding alterations within the putative PEX19-binding motifs of DENV C would negatively affect their binding to PEX19 (Figure 2C). Replacing either the consecutive residues lysine-leucine at positions 45 and 46 (K45A and L46G, referred to as KL45) or arginine-phenylalanine at positions 55 and 56 (R55A and F56G, referred to as RF55) did not affect the interaction between DENV C and PEX19, but a combination of these mutations (K45A, L46G, R55A and F56G, referred to as KL45/RF55) resulted in the loss of the ability of DENV C to bind PEX19.

Finally, in addition to the overexpression experiments using ^SBP^PEX19, we examined whether the identified PEX19-binding motif of DENV C is also necessary for binding to endogenous PEX19. As shown by coimmunoprecipitation using anti-PEX19 antibodies, only the WT DENV C was able to interact with PEX19 (Figure 2D).

Together, these results show that DENV C contains a PEX19-binding domain and reveal the importance of amino acids with positively charged side chains at residues 45 and 55 for enabling the interaction with PEX19.

### 3.3. The PEX19-Binding Motif Is Conserved in ZIKV C

Since we confirmed the existence of a PEX19-binding motif in DENV C and our bioinformatic analysis also predicted a putative PEX19-binding motif in ZIKV C (Figure 2B), which has previously been reported to interact with PEX19 [19], we aimed to confirm the existence of a PEX19-binding motif in ZIKV C to understand whether the PEX19-binding determinants are conserved between DENV and ZIKV C.

**Figure 3:**
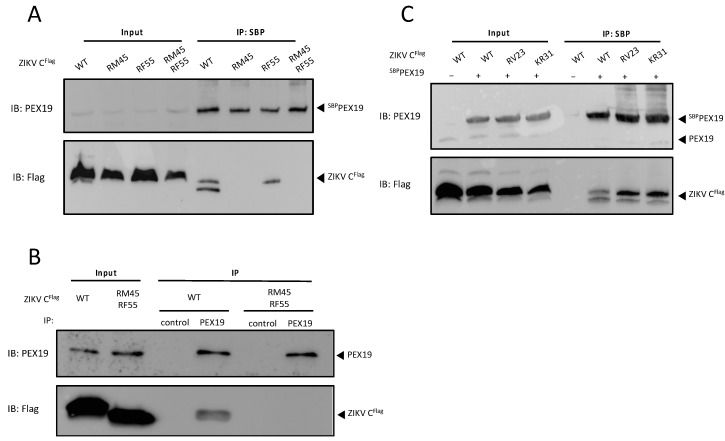
ZIKV capsid protein binds PEX19 via a conserved PEX19-binding motif. (**A**,**C**) ^SBP^PEX19 was co-expressed in HEK293T cells together with the ZIKV C^Flag^ WT construct or versions with mutations (RM45, RF55, RM45/RF55, RV23, or KR31), as indicated. ^SBP^PEX19-ZIKV C^flag^ complexes were affinity purified using streptavidin agarose bead and analyzed by immunoblotting using PEX19- or flag-specific antibodies, respectively. (**B**) The ZIKV C WT, or the C variant RM45/RF55 was expressed in HEK293T cells. Protein complexes were affinity purified with PEX19-specific antibodies. An unrelated antibody was used as negative control for the co-IP (control). Samples were subsequently analyzed by immunoblotting using PEX19- or flag-specific antibodies, respectively. An aliquot (15%) of the input is shown. The amount loaded for input represents 15% of the amount loaded for the IP for all western blots.

Using the approach described above, three ZIKV C mutants were generated by replacing either the consecutive residues arginine-methionine at positions 45/46 (R45A and M46G, referred to as RM45) or arginine-phenylalanine at positions 55/56 (R55A and F56G, referred to as RF55), or combining all modifications (R45A, M46G, R55A, and F56G, referred to as RM45/RF55). PEX19 binding was assessed by ^SBP-^PEX19 pull-down assay. We confirmed published data indicating an interaction between ZIKV C and PEX19 [19], and similar to the results obtained with DENV C, the ZIKV C variant containing the combined mutations failed to interact with PEX19 (Figure 3A). Interestingly, replacing the consecutive residues arginine-methionine at positions 45/46 of ZIKV C protein with alanine-glycine inhibited the binding of PEX19 to the C protein (Figure 3A), while a similar exchange within the DENV C did not interfere with its PEX19 interaction (Figure 2C). Furthermore, the disruption of PEX19 binding by mutations in residues 45, 46, 55 and 56 of ZIKV C was confirmed by co-immunoprecipitation with endogenous PEX19 (Figure 3B).

Additionally, to confirm the specificity of our findings, we generated two further ZIKV C variants with mutations outside the predicted PEX19-binding motif but with identical amino acid replacements to those found to disrupt PEX19 binding. Amino acids with electrically charged side chains (R23, K31) were exchanged by alanine, while the following amino acid was replaced by glycine (R23A/V24G, referred to as RV23; and K31A/R32G, referred to as KR31). The pull-down assays show that both variants ZIKV C RV23 and KR31 retain their ability to bind PEX19 (Figure 3C).

Taken together, these results suggest that a conserved PEX19 binding motif, which contains key amino acids with positively charged side chains, is present in both DENV and ZIKV C. However, residues within this conserved binding motif might make different contributions to the PEX19 interaction.

### 3.4. ZIKV C and DENV C Subcellular Localization Is Independent of PEX19

**Figure 4:**
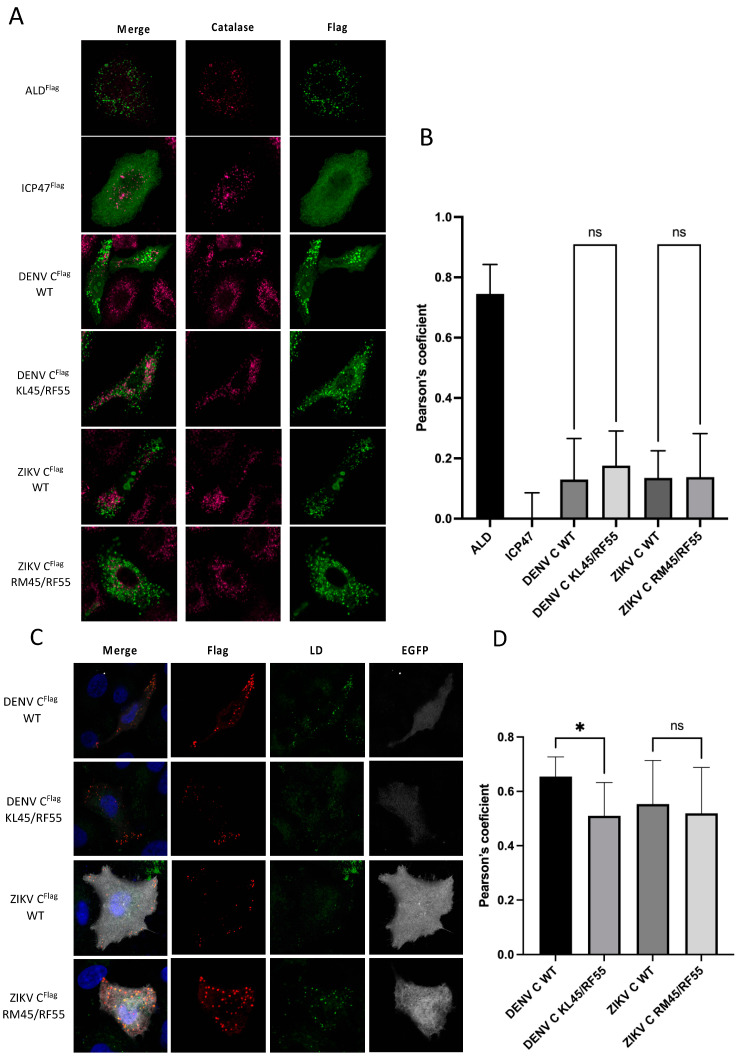
PEX19 is not involved in C transport to organelles. (**A**) A549 cells were transfected with plasmids encoding for the human peroxisomal membrane marker ALD (ALD^flag^), the HSV-1 protein ICP47 [35] (ICP47^flag^), and the WT or mutated variants of the DENV (DENV C^flag^ KL45/RF55) and ZIKV (ZIKV C^flag^ RM45/RF55) C proteins. 24 h post-transfection, cells were fixed and stained for indirect immunofluorescence using catalase-specific and flag-specific antibodies. (**B**) Colocalization analysis of flag-positive structures and catalase using Pearson’s coefficient. Statistical analysis was carried out using an ordinary one-way ANOVA with Sidak’s multiple comparisons test (*n* > 7; ns = non-significant). (**C**) A549 cells were transfected with pIRES2-EGFP derived plasmids encoding for WT or mutated variants of the ZIKV or DENV C proteins (ZIKV C^flag^ WT, DENV C^flag^ WT, ZIKV C^flag^ RM45/RF55 or DENV C^flag^ KL45/RF55, respectively). At 48 h post-transfection, cells were processed for indirect immunofluorescence using anti-flag antibody for the detection of C proteins (red). Cells were also stained with LipidTOX deep red for lipid droplets detection (indicated in green). Nuclei were stained by DAPI and overlaid with anti-mouse 594 and LipidTOX deep red signals (merge). Intrinsic EGFP fluorescent is shown in gray. (**D**) Colocalization analysis of flag tag with lipid droplets using Pearson’s coefficient. Statistical analysis was carried out using an ordinary one-way ANOVA with Sidak’s multiple comparisons test (*n* > 9; ns = non-significant, * = *p* < 0.05).

PEX19 is a cytosolic chaperone responsible for escorting PMPs, which contain a PEX19 binding-motif and at least one transmembrane domain, to peroxisomal membranes [20,21,22,23,25,26,31,34]. Although we show that DENV C and ZIKV C bind to PEX19 via a conserved PEX19 binding-motif, the role of this interaction remains unknown. To understand whether interaction with PEX19 would lead to localization of C to peroxisomes via the targeting receptor and chaperone PEX19, peroxisomal localization of WT or mutant ZIKV C or DENV C was assessed by fluorescence microscopy. As expected, ALD colocalized with peroxisomes, visualised by catalase staining, while the HSV-1 ICP47 protein, which does not bind PEX19, did not (Figure 4A). Quantification of colocalization shows that none of the overexpressed C proteins colocalized with peroxisomal structures (Figure 4B).

Recently, PEX19 was found to not only be responsible for the transportation of integral PMPs to peroxisomes, but also for being a key element in targeting of proteins to lipid droplets (LDs) [36]. LDs are endoplasmic reticulum (ER)-derived organelles that store energy as triglycerides, which are surrounded by a phospholipid monolayer. LD membrane proteins are embedded into this monolayer in a monotopic hairpin topology [37]. Similar to mature flaviviral C proteins, they do not contain a bilayer-spanning transmembrane segment, and therefore are unusual PEX19 substrates. Interestingly, recent evidence suggests that the mature C proteins of ZIKV and DENV accumulate on the surface of LDs [38,39,40], hence it is tempting to speculate that interaction of the C proteins with PEX19 might be important for facilitating their sorting to these ER-derived organelles. This hypothesis is supported by the fact that amino acids L50 and L54 of the DENV C protein, which are both localized within the identified PEX19-binding motif (Figure 2B), are specifically necessary for correct targeting of the C protein to LDs [40]. Therefore, we assessed whether other C protein variants that lost their ability to interact with PEX19 (ZIKV C RM45/RF55 and DENV C KL45/RF55) likewise fail to be sorted to LDs. As expected, WT version of both DENV and ZIKV C localized to lipid droplets (Figure 4C). No changes to this lipid droplet localization were observed when comparing ZIKV C RM45/RF55 with the WT (Figure 4C,D). However, there was a small but significant decrease in LD localization of DENV C KL45/RF55 (Figure 4C,D). Nevertheless, the DENV C protein still retained most of its ability to localize at the LD membrane, which would not be expected to occur if PEX19 was solely responsible for its transportation to these organelles. Therefore, these results suggest that binding to PEX19 is not an essential prerequisite for the proper targeting of ZIKV and DENV C to LDs or peroxisomes.

### 3.5. ZIKV Capsid Protein Reduces Peroxisome Abundance

Recent studies revealed that in DENV-, ZIKV-, or WNV-infected cells, the number of peroxisomes is decreased [17,18,19]. Moreover, the expression of either DENV [17] or ZIKV [19] C protein alone has been found to lead to a significant loss of peroxisomes in A549 or U251 cells, respectively. You et al. proposed that depletion of peroxisome numbers is mediated by the C-PEX19 interaction [17]. In order to test this hypothesis, we overexpressed either WT ZIKV C or the KL45/RF55 variant in A549 cells and assessed peroxisome abundance through fluorescent microscopy.

**Figure 5:**
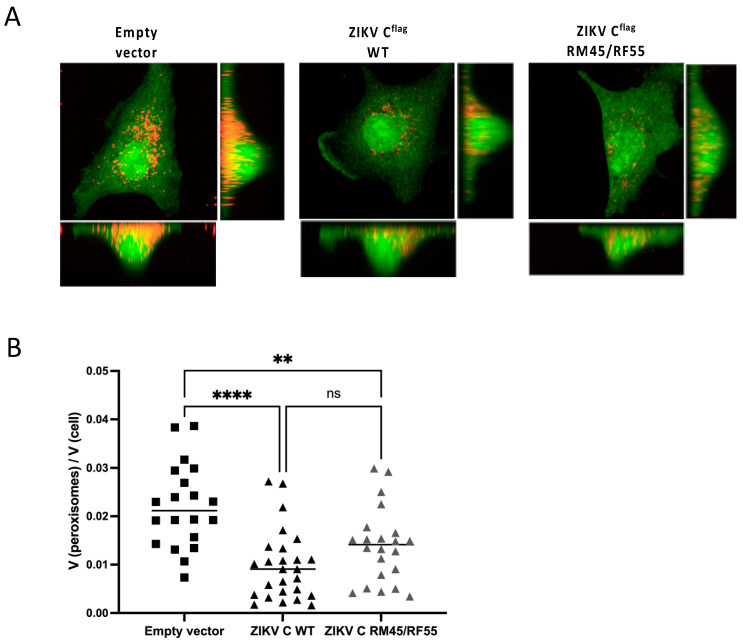
Expression of ZIKV capsid proteins causes loss of peroxisomes. (**A**) A549 cells were transfected with pIRES2-EGFP-derived plasmids encoding the ZIKV C WT or mutated variant (ZIKV C RM45/RF55) of the ZIKV C protein, or the empty vector as negative control. At 72 h post transfection, cells were processed for indirect immunofluorescence using mouse anti-PMP70 antibodies for the detection of peroxisomes (in red). Images show the top and side view Z stack reconstruction of a single cell. (**B**) Z-stack images of GFP-positive cells were acquired in 0.2 μm intervals, and peroxisome abundance analysis was performed using the total volume of peroxisomes:total volume of the cell ratio. Statistical analysis was carried out using an ordinary one-way ANOVA with Tukey’s multiple comparisons test (*n* > 20; ns = non-significant, ** = *p* < 0.01, **** = *p* < 0.0001).

We observed that, similarly to what has been reported with DENV C or WNV C, overexpression of WT ZIKV C results in a significant decrease in peroxisome abundance, compared to cells overexpressing an empty vector (Figure 5A). Strikingly, expression of ZIKV C RM45/RF55, which lacks the ability to interact with PEX19, also resulted in a significant loss of peroxisomes in A549 cells (Figure 5A,B). Although a small difference in peroxisome abundance was seen in cells expressing ZIKV C RM45/RF55 compared to cells expressing WT ZIKV C, this difference was not statistically significant. These results show that the absence of C-PEX19 binding does not restore peroxisome abundance to levels comparable to cells expressing the empty vector, and shows that the C-PEX19 interaction is not essential for mediating reductions in peroxisome abundance.

### 3.6. ZIKV C Impairs Peroxisome Biogenesis Independently of PEX19 Binding

Peroxisomes are dynamic organelles and their abundance is regulated by two processes: biogenesis, the generation of new peroxisomes; or by pexophagy, a selective autophagy that targets peroxisomes for degradation [41,42]. Both ZIKV C and DENV C have been shown to reduce peroxisome abundance [17,19], however it remains unknown whether these changes are due to decreased peroxisomal biogenesis or increased pexophagy. Thus, we next assessed whether these proteins directly disturb peroxisomal de novo synthesis.

**Figure 6:**
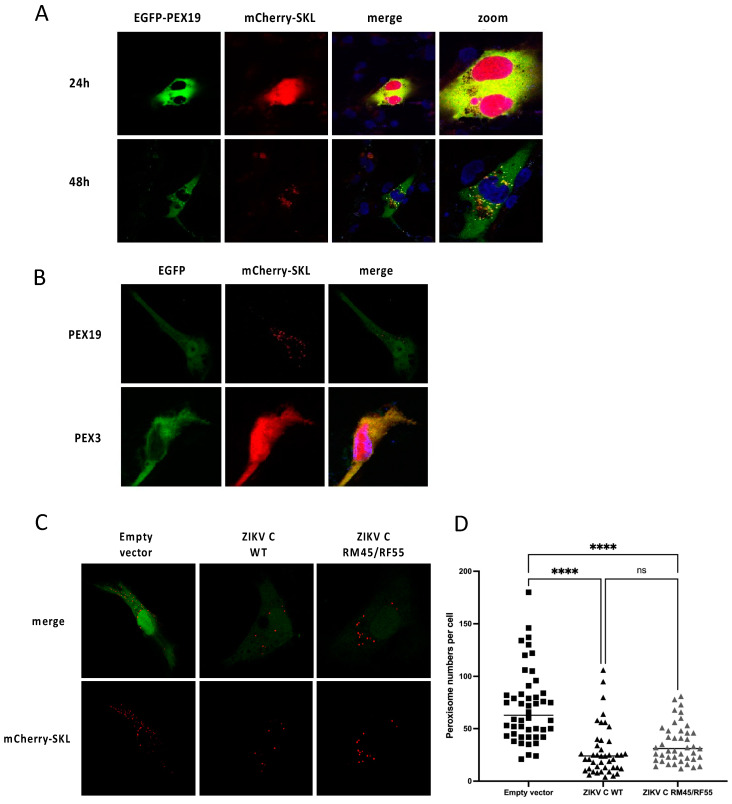
ZIKV capsid impairs peroxisome biogenesis. (**A**) Time course of peroxisomal rescue in PEX19-deficient fibroblasts. PEX19-deficient patient-derived human fibroblast were transfected with the plasmid pNoGFP-EGFP-SBP-PEX19 (EGFP-PEX19) to rescue peroxisomal biogenesis. The co-expressed mCherry-SKL serves as a peroxisomal lumenal marker. Cells were fixed and analyzed by intrinsic EGFP and mCherry fluorescence either 24 h or 48 h post-transfection. Nuclei were stained using DAPI (blue). (**B**) Peroxisomal rescue is induced by PEX19. PEX19-deficient fibroblasts were transfected with the plasmid pIRES2-mCherry-SKL/PEX19 or pIRES2-mCherry-SKL/PEX3. In parallel, cells were co-transfected with the empty pIRES2-EGFP vector. At 48 h post-transfection, cells were fixed and analyzed for intrinsic GFP and mCherry fluorescence. (**C**) PEX19-deficient patient-derived human fibroblast were transfected with the plasmid pIRES2-mCherry-SKL/PEX19 to rescue peroxisomal biogenesis by the human PEX19 WT cDNA, as well as the peroxisomal lumenal marker protein mCherry-SKL. In parallel, cells were co-transfected with pIRES2-EGFP derived plasmids encoding the WT or mutated variant of the ZIKV C protein (ZIKV C RM45/RF55), or the empty pIRES2-EGFP plasmid as negative control. At 48 h post-transfection, cells were fixed and analyzed for intrinsic GFP and mCherry fluorescence. (**D**) Peroxisomal abundance in GFP-positive cell was quantified using ImageJ. Statistical analysis was carried out using an ordinary one-way ANOVA with Tukey’s multiple comparisons test (*n* > 35; ns = non-significant, **** = *p* < 0.0001).

Patient-derived skin fibroblast with a defective *PEX19* gene do not contain any functional peroxisomes [4]. However, expression of the WT *PEX19* cDNA in these *PEX19*-deficient fibroblasts restores the biogenesis of functional peroxisomes [4,28]. Due to the fact that these PEX19-defective fibroblast cell lines do not contain any morphologically recognizable peroxisomes [4], the restoration of functional peroxisomes occurs slowly. Peroxisomes were first detectable at 48 h post transfection, as revealed by a time course experiment where cells were transfected with two different plasmids to co-express a GFP-tagged PEX19 variant and a peroxisomal marker protein mCherry-SKL, in which “SKL” (serine-lysine-leucine) is the amino acid sequence that serves as the peroxisomal targeting signal [29] (Figure 6A). A 48 h delay in peroxisome rescue is expected and has been previously observed in similar experiments with *PEX3*-deficient [5] or *PEX16*-deficient [43] cell lines.

Next, we generated a pIRES2-derived plasmid that allows the translation of PEX19, as well as the peroxisomal marker protein mCherry-SKL [29], from a single bicistronic mRNA. *PEX19*-deficient fibroblasts were transiently transfected with the pIRES2-mCherry-SKL/PEX19 plasmid, and the reoccurrence of functional peroxisomes was monitored by the appearance of mCherry-SKL positive structures at 48 h post-transfection (Figure 6B), while the plasmid pIRES2-mCherry-SKL/PEX3, which encodes for the peroxisomal biogenesis gene *PEX3*, did not restore peroxisomal structures (Figure 6B).

Then, to assess the reoccurrence of functional peroxisomes in *PEX19*-deficient fibroblasts in the presence of ZIKV C, cells were transfected with a plasmid to co-express PEX19 and mCherry-SKL, and another to express either ZIKV C WT or ZIKV C RM45/RF55. Notably, de novo peroxisome biogenesis was significantly delayed at 48 h post-transfection in the presence of ZIKV C WT, suggesting that the reduction of peroxisome abundance by ZIKV C is, at least in part, due to impairment of de novo peroxisome biogenesis. Interestingly, a similar delay in peroxisome reconstitution was observed in cells expressing the mutated variant ZIKV C RM45/RF55 (Figure 6C,D), which does not bind PEX19, suggesting that the impairment in peroxisome biogenesis is not mediated by the interaction between PEX19 and ZIKV C. Thus, ZIKV C may interfere with peroxisomal abundance and biogenesis by a yet unknown, PEX19-independent mechanism.

## 4. Discussion

Several viruses have been found to modulate peroxisomes either to suppress peroxisome-mediated antiviral immunity or to exploit the peroxisomal metabolism for viral replication [8]. It has been proposed that viruses, including the flaviviruses WNV, DENV and ZIKV, impair peroxisome biogenesis as a strategy to suppress peroxisome-dependent innate immune responses in order to enhance viral replication [17,19]. On the other hand, reports show that the flavivirus life cycle is impaired when peroxisomes numbers are reduced [17,18], which contradicts this hypothesis. Here, we aimed to better understand the role of peroxisomes and PEX19 in the DENV and ZIKV life cycle.

By infecting two patient-derived cell lines lacking peroxisomes (Δ*PEX19* [4] and Δ*PEX3* [5]) with DENV-2, we showed that DENV replication is impaired in the absence of peroxisomes compared to a control cell line (HFFF). This impairment in DENV-2 replication mirrors the previously published data showing that ZIKV replication is reduced by 1–2 log in the same *PEX19*-deficient and *PEX3*-deficient cell lines under similar growth conditions when compared to wild-type fibroblasts [18]. Until now, the importance of peroxisomes in the DENV life cycle has only been studied in one previous study, which used A549 cells in which PEX19 expression was only transiently decreased by 60% using siRNAs [17]. Furthermore, no information about peroxisome abundance was provided in this study. Nonetheless, similarly to our data from cells completely deficient in peroxisomes, the researchers also observed an impairment of DENV replication compared to cells without siRNA treatment [17]. Taken together, these results suggest a pro-viral role of peroxisomes in the life cycle of flaviviruses. A pro-viral role of peroxisomes was recently reported for KSHV, as peroxisomes in combination with MAVS were shown to be involved in the stabilization of the KSHV oncoprotein vFLIP, thereby promoting KSHV latency [9]. In addition, HCMV and HSV-1 induce the biogenesis of peroxisomes as well as unique morphological changes to these organelles in order to support their replication [44]. It was hypothesized that HCMV and HSV-1 infection initiates peroxisomal growth and fission in order to use peroxisomal lipid metabolism for the assembly of infectious particles [44]. While it remains to be seen how peroxisomes are mechanistically linked to enhanced DENV replication, our findings highlight the importance of peroxisomes in flavivirus biology.

Several flavivirus C proteins have been reported to bind PEX19, a key player in peroxisome biogenesis [4]. We identified a PEX19 binding motif in the ZIKV and DENV C proteins, which consists of functionally essential basic and hydrophobic amino acids. Exchange of these conserved residues with neutral residues within this common binding motif, which is shared with cellular PMP cargos of PEX19, inhibited the interaction between the mature C protein and PEX19. Interestingly, in a published analysis comparing the amino acid sequence of 20 mosquito-borne flavivirus C proteins, residues 45 and 55—key residues for PEX19 interaction—were found to be conserved in all flaviviruses [45]. Interaction of cellular proteins with PEX19 additionally relies on the presence of a PEX19 binding domain and a transmembrane segment in cargo proteins. Recently, a study showed that UBX domain-containing protein 8 (UBXD8), a protein that lacks transmembrane segments, interacts with PEX19. However, this protein contains a hydrophobic hairpin domain that allows post-translational insertion of UBXD8 into lipid droplets [36]. To our knowledge, mature DENV and ZIKV C proteins are the first PEX19 interacting partners that do not require a transmembrane segment or hairpin domain for their interaction with PEX19. Although mature ZIKV and DENV C do not contain a transmembrane segment or hairpin domain, both have a hydrophobic region in the α2 helix [45,46,47], which could function as a “transmembrane segment analogue” for PEX19 recognition. Analysis of hydrophobicity also revealed a conserved hydrophobic α2 helix among all analysed flavivirus C proteins [45]. Thus, it is conceivable that the interaction between PEX19 and C might not be exclusive to DENV, ZIKV, and WNV, but rather present in several viruses of the *Flavivirus* genus. If this were true, PEX19 would likely play a key role in the flavivirus life cycle.

PEX19′s main role is to escort PMPs from the cytosol to the peroxisomal membrane by recognizing their PMP targeting signal and binding to the hydrophobic domains [20,21,22,23,25,26,31,34]. PMPs are then transported and inserted into the peroxisomal membrane with the help of the PMP import machinery: PEX19, PEX3 [48], and PEX16 [49]. This chaperone and transport role of PEX19 is exploited by diverse viruses. The tomato bushy stunt virus (TBSV) p33 protein and the HCMV vMIA protein were both shown to interact with PEX19 in order to be delivered to peroxisomes [13,50]. ZIKV C and DENV C are known to localize to LD [36,37,38,44], and in the nucleolus [39,51,52,53,54], but a peroxisomal localization of ZIKV C or DENV C was never described. We also did not observe a colocalization of C and peroxisomes (Figure 4A), demonstrating that although DENV and ZIKV C proteins bind PEX19 they are not transported to peroxisomes. These results are supported by You et al., who also did not observe localization of C in peroxisomes during viral infection with DENV or WNV, or when C was expressed in isolation [17]. Recently, PEX19 was also shown to mediate the localization of proteins to LDs. PEX19 mediates the insertion of UBXD8, a protein that belongs to a subset of LD-targeted proteins, into ER subdomains where PEX3 is present that are subsequently partitioned into the LD monolayer [36]. It has been previously reported by Samsa et al. that mutations in residues L50 and L54 of DENV C, which are located within our predicted PEX19 binding motif, are necessary for the accumulation of C in LDs [38]. This led us to hypothesize that the C-PEX19 interaction could have a possible role in the targeting of C to LDs. However, our results show that ZIKV C still localizes to LDs when its interaction with PEX19 is disrupted through independent mutations in its PEX19 binding motif, therefore demonstrating that the transport of ZIKV C and DENV C to LDs is not mediated by PEX19. The observation that DENV and ZIKV C are not transported to organelles that canonically import proteins through PEX19 is somewhat unexpected and makes the C-PEX19 interaction unusual compared to other viral and cellular binding partners of PEX19.

Previous studies have suggested that flavivirus mature C proteins directly sequester the biogenesis chaperone PEX19, thereby impeding the binding of PMPs to PEX19. It is hypothesized that this leads to an impairment of peroxisome biogenesis, reducing the number of peroxisomes in the cell, which ultimately results in a weakened interferon response [17]. This model has been supported by the observation that increasing peroxisomal abundance by over-expression of the biogenesis factor PEX11B was shown to inhibit ZIKV replication [19]. However, we here showed that the presence of ZIKV C leads to a reduction in peroxisome abundance that is not mainly mediated by the interaction with PEX19, since addition of a mutation that disrupts PEX19 binding results in a similar decrease in peroxisome abundance. This is supported by the observation that ZIKV C impairs de novo peroxisome biogenesis regardless of its ability to interact with PEX19, suggesting that the main mechanism for the observed reduction of peroxisome numbers is not the appropriation of PEX19 by the ZIKV C protein. What influence the C-PEX19 interaction has on peroxisome abundance in the context of viral infection, and whether multiple redundant mechanisms by which ZIKV C proteins downregulate peroxisomes exist, remains to be determined. Although the molecular mechanisms by which ZIKV C proteins lead to a reduction of peroxisomes remains unknown, here we demonstrated for the first time that it is, at least in part, through impairment of de novo biogenesis. How these observations translate to other flavivirus C proteins, including DENV C, remains to be determined.

The C proteins of flaviviruses play an important role in virus assembly. They localize to the ER membrane and recruit the viral genome, forming the nucleocapsid, which subsequently buds into the ER, which contains the viral envelope and membrane proteins [55]. A mechanism by which C associates to ER membranes for viral replication and assembly, physically disrupting the “budding off” of newly made pre-peroxisomes at the ER, could explain our findings showing that ZIKV C impairs the de novo peroxisome biogenesis. The role of peroxisomes in flavivirus infection remains poorly understood and therefore should be further explored. While we and other groups have shown that complete and partial peroxisome loss results in impaired flavivirus replication [17,18], suggesting a pro-viral role of peroxisomes in infection, another study showed that an increase in peroxisome numbers, by overexpression of PEX11, has an anti-viral effect against ZIKV infection, likely due to an increase in the peroxisome-dependent innate immune response [19]. Thus, it is possible that flaviviruses modulate peroxisome numbers (potentially in a temporal manner) to minimize the peroxisome-dependent antiviral response while maintaining an appropriate number of peroxisomes needed for viral replication.

In conclusion, our findings challenge the current model of how flavivirus C proteins downregulate peroxisomal abundance, by showing that, at least for ZIKV, this occurs independently of the C-PEX19 interaction. We also find that the C-PEX19 interaction does not affect the canonical functions of PEX19, revealing an open question regarding the functional consequences of the conserved C-PEX19 interaction. Nevertheless, our data highlight the important role that peroxisomes play in flavivirus replication. Gaining deeper insights into flavivirus-peroxisome interactions may therefore reveal new approaches to developing antiviral therapies against this important group of pathogens. Finally, by defining the PEX19-binding motif within the C proteins of two flaviviruses, we identify the mature C protein as having unique PEX19-binding determinants compared to other PEX19 cargos. This knowledge, and the valuable tools we developed, will therefore also enable the more general molecular determinants of PEX19 interactions to be interrogated, with broader consequences for host cell biology.

## Data Availability

The data presented in this study are openly available in FigShare at [doi: https://doi.org/10.6084/m9.figshare.c.5811113.v1].
